# The Co-creation and Feasibility of a Compassion Training as a Follow-up to Mindfulness-Based Cognitive Therapy in Patients with Recurrent Depression

**DOI:** 10.1007/s12671-017-0783-1

**Published:** 2017-08-05

**Authors:** Rhoda Schuling, Marloes Huijbers, Hetty Jansen, Renée Metzemaekers, Erik Van Den Brink, Frits Koster, Hiske Van Ravesteijn, Anne Speckens

**Affiliations:** 1 Radboudumc Centre for Mindfulness, P.O. Box 9101, (huispost 966), 6500 HB Nijmegen, The Netherlands; 2 MBCL Training & Therapy, http://www.mbcl.nl; 3 Centre for Integral Psychiatry, Hereweg 80, 9725 AG Groningen, The Netherlands

**Keywords:** Mindfulness, Self-compassion, Recurrent depression, Qualitative study, Co-creation

## Abstract

The aim of this study was to assess the feasibility, acceptability and preliminary effectiveness of Mindfulness***-***Based Compassionate Living (MBCL) as a follow-up intervention to Mindfulness Based Cognitive Therapy in adults with recurrent depression. We conducted an uncontrolled study in 17 patients with recurrent depression, in two successive groups. The first group contained novices to compassion training (*N* = 14); in the second group, ten of these participated again, in addition to three new participants (*N* = 13). The overall group contained 15 females and 2 males, aged between 37 and 71. The MBCL program was qualitatively evaluated using post-intervention focus group interviews in both groups. In addition, self-report questionnaires assessing depressive symptoms, worry and both self-compassion and mindfulness skills were administered before and after MBCL. No patients dropped out of the intervention. Average attendance was 7.52 (SD 0.73) out of eight sessions. Helpful elements were theory on the emotion regulation systems, practicing self-compassion explicitly and embodiment of a compassionate attitude by the teachers. Unhelpful elements were the lack of a clear structure, lack of time to practice compassion for self and the occurrence of the so-called back draft effect. We adapted the program in accordance with the feedback of the participants. Preliminary results showed a reduction in depressive symptoms in the second group, but not in the first group, and an increase in self-compassion in both groups. Worry and overall mindfulness did not change. MBCL appears to be feasible and acceptable for patients suffering from recurrent depressive symptoms who previously participated in MBCT. Selection bias may have been a factor as only experienced and motivated participants were used; this, however, suited our intention to co-create MBCL in close collaboration with knowledgeable users. Examination of the effectiveness of MBCL in a sufficiently powered randomised controlled trial is needed.

**Electronic supplementary material:**

The online version of this article (doi:10.1007/s12671-017-0783-1) contains supplementary material, which is available to authorized users.

## Introduction

Major depressive disorder (MDD) is one of the most prevalent psychiatric disorders. It is characterized by high relapse rates (Mueller et al. 1999; Solomon et al. 2000), partly due to persistence of residual symptoms after remission (Hardeveld et al. 2010). Given the increasing risk of relapse after each successive episode, prevention of relapse is as important as acute treatment (Hardeveld et al. 2010). To address the need for psychological interventions targeting relapse prevention, Segal, Williams and Teasdale developed Mindfulness-Based Cognitive Therapy (MBCT; (2000). A meta-analysis (Kuyken et al. 2016) showed that MBCT for patients with recurrent depression in remission resulted in a reduction of the risk of a relapse/recurrence of 31%. A growing number of studies indicate that MBCT may also be effective in decreasing current depression (Strauss et al. 2014). Van Aalderen et al. (2015) reported effectiveness of MBCT to be comparable in both remitted and currently depressed patients substantiating effectiveness and acceptability of MBCT as acute treatment of depression.

Though these results are encouraging, even after MBCT room for improvement remains considerable (Piet and Hougaard 2011) and mild levels of depression (average BDI score of 10) remain present in many patients (Van Aalderen et al. 2015). It is therefore necessary to explore ways to further improve outcomes for recurrently depressed patients. A closer look at working mechanisms of MBCT yields insight in how to proceed: Kuyken et al. (2010) showed that the effect of MBCT on relapse/recurrence was mediated by increased self-compassion and mindfulness. Both significantly predicted depression levels 13 months after treatment: patients who reported an increase in mindfulness skills or self-compassion had lower rates of depressive symptoms. It seems one of the evident options to explore in improving outcome for recurrently depressed adults is self-compassion.

Mindfulness-Based Compassionate Living (MBCL) program was designed as a follow-up intervention for patients who have already attended MBCT or MBSR (Van den Brink and Koster 2012; Van den Brink and Koster 2015). Van den Brink and Koster (2015) identified two components of compassion: “(1) developing the willingness and courage to turn towards suffering both in oneself and others rather than turning away and (2) dedicating oneself to acquiring the wisdom and skills and engaging in the appropriate actions for alleviation and prevention of suffering” (p. Xvii). Amongst others, the MBCL is inspired by Compassion Focused Therapy (Gilbert 2009), with its focus on use in clinical settings, and by Neff and Germer’s Mindful Self-Compassion program for non-clinical populations (2013). Preliminary results of these programs indicated people may benefit from a training in self-compassion (Gilbert 2009; Gilbert and Procter 2006; Neff and Germer 2013). Lack of self-compassion was both linked to negative self-esteem and self-criticism, as well as the maintenance and recurrence of depression (Gilbert 2009; Neff 2003). According to Neff’s (2013) trial with the mindful self-compassion program, significantly larger increases in self-compassion, mindfulness and wellbeing in intervention participants ere found compared to a control group. In a recent meta-analysis, compassion was indicated as an important explanatory variable in understanding mental health and resilience (MacBeth and Gumley 2012).

MBCL is a group-based intervention which consists of eight 2.5-h sessions once every fortnight and a silent day. It uses a format similar to MBCT, combining central practices, inquiry and didactic teaching. Theory focuses on the following aspects: (1) (dis)balance of emotion regulation systems (*threat*, *hunt* and *rest and digest* system); (2) instinctive stress reactions (fight-flight-freeze) and more nourishing stress responses (tend-befriend); (3) three modes of operation (threat mode, competitive mode and compassion mode), with emphasis on the inner critic or bully as a particular manifestation of the threat mode or system; (4) the back draft effect (how compassion practice to self and others may give space for old pain to resurface, causing the participant to feel overwhelmed and, often, discouraged); (5) relational qualities of compassion; (6) processes of over-and de-identification from a compassionate perspective; and finally (7) common humanity and the four life companions (kindness, compassion, joy and equanimity). Theory in each session is supported by accompanying practices which often include imagination, i.e. imagining a safe haven for oneself or imagining a compassionate companion. The Metta or befriending practice is gradually expanded over the course of the sessions: in the first four sessions, emphasis is on self-compassion, after which the compassion practice expands to include compassion for other people.

The importance of self-compassion within the MBCT program has also been underscored by Segal et al. (2012), who stated: “... one of the most important things people learn from an MBCT program is kindness and self-compassion. We regard this as fundamental.” (p.137). However, it may be the case that particularly patients with recurrent depression need more explicit instructions and additional support to develop a compassionate attitude to both self and others, as they often are highly self-critical and plagued by feelings of shame, guilt and inferiority (Gilbert et al. 2008, 2012; Gilbert and Procter 2006). In contrast to the more implicit teaching of compassion in MBCT, cultivating compassion is the primary focus of MBCL. Throughout the entire curriculum, the invitation is to practice kindness and compassion to self and others in the midst of suffering. This is in fact one of the main differences between MBCL and MBCT. MBCL deliberately gears towards focus on unpleasant experiences. Investigation of feasibility is therefore warranted, which in addition to this exposure to the difficult might shed light on the influence of the more varied practices offered in MBCL and the fact that many MBCL practices make use of participants’ imaginative ability.

To ensure participants have had opportunity to practice observing and de-identification with thoughts and emotions, before attending to the more difficult ones, MBCL has been designed as a follow-up to MBCT. Practices such as the ‘safe haven’ and ‘compassionate companion’ are specifically designed as a further support for this. So far, the MBCL program has been evaluated in a (small) heterogeneous clinical sample (Bartels-Velthuis et al. 2016), but not in a recurrently depressed population specifically.

The aim of our study was to investigate the feasibility and acceptability of the MBCL program as a follow-up intervention after MBCT in patients with recurrent depression, by qualitatively assessing helpful and unhelpful elements of the program. Furthermore, we examined the preliminary effectiveness of the MBCL to reduce depressive symptoms as well as worry, a common type of perseverative negative thinking in patients suffering from recurrent depression which often induces or maintains depressive symptoms. We also examined preliminary effectiveness on self-compassion and mindfulness skills.

## Method

### Participants

Regarding who should participate in co-creation of technology services, Franke et al. (2006) claimed that users who are able to co-create should have cutting edge knowledge within the area; thus, only consumers who are “leading users” should be involved. We therefore sought highly motivated patients with recurrent depression, who had previously participated in MBCT, who were willing to invest time and effort in attending and evaluating the MBCL program and able to offer an informed opinion. We recruited possible participants at the regular reunion meetings offered at the Radboudumc Centre for Mindfulness in Nijmegen, the Netherlands. Inclusion criteria were minimum age of 18 and diagnosis of recurrent depression according to the Diagnostic and Statistical manual of Mental disorders (4th edition) criteria (First et al. 1996). Most patients had participated in previous research for which the interview data was known. For those who had not, we conducted Mini International Neuropsychiatric Interviews (MINIs; (Sheehan et al. 1998). Patients with past (hypo) manic or psychotic episodes or recent alcohol and/or drug abuse (last 12 months) were excluded.

Fourteen patients participated in the first MBCL course. Ten of them and three additional patients participated in the second course. Average attendance rate was 7.4 (first course) and 7.6 (second course) out of eight sessions. The majority of the participants were female (*N* = 15, 87%) and the average age was 53.4 (SD 9.3). Attendance rate of the MBCT is known for seven out of 17 participants: this was eight sessions for all of them. For specifics of each group, see Table 1.

**Table 1:** Gender, age and time lapse since MBCT of each group

	Group 1 (*N* = 14)	Group 2 (*N* = 13)	
		Doubles (*N* = 10)	Novices (*N* = 3)
Female (*N*)	13	9	2
Age (SD)	56 (9.8)	58.7 (5.7)	57.8 (2.1)
Age range	37–71	46–66	56–60
Time lapse since MBCT (months)	32.1	47.3	54

### Procedure

We set up a pilot study to develop and evaluate MBCL as a follow-up intervention to MBCT in adults with recurrent depression. After the training, we conducted a focus group interview, after which necessary adaptations were made to the MBCL program. By involving patients in the development of the program in this way, we essentially opted for co-creation of a new format. Though very little described or researched in the field of Psychology, in technology development, co-creation refers to collaboration with customers for the purpose of innovation and has become a foundational premise of the service-dominant logic (Lusch et al. 2007). In this context, the basis for the collaboration is the experiences that a customer has gained when using a company’s product or service (Vargo and Lusch 2004), in order to ascertain the value of that product or service and unearth latent customer needs that the service should address.

We recruited participants until a first group could be formed (*N* = 14). The MBCL was offered in accordance with the curriculum of the original developers (Van den Brink and Koster 2012). Patients were invited to practice at home for about 30 min on a daily basis, supported by CDs, and to keep a record of their experiences. The intervention was taught by two teachers (HJ and RM) who both meet the advanced criteria of the Association of Mindfulness-Based Teachers in the Netherlands and Flanders (which correspond to the Good Practice Guidelines for teaching mindfulness-based courses by the UK Network for Mindfulness-Based Teacher Training Organizations). In addition, both were trained in the MBCL program by the developers (Van den Brink and Koster).

Following the last session of the first group, a focus group interview was held on facilitators and barriers, i.e. helpful and unhelpful elements of the course, led by an experienced researcher (AS) who had not been involved in the training. Also present were both teachers (HJ and RM) and a junior researcher taking notes (RS). Focus groups started with explaining confidentiality and the explorative nature of the interview. Questions were asked in an open non-directive manner, allowing participants to speak freely about their experiences. The research question addressed in the interview was “What was helpful in the training and what difficult?” and “What improvements, if any, could be made?”. The duration of the interview was one-and-a-half hours. The interview was audio and videotaped.

Self-report questionnaires on depressive symptoms, worry, mindfulness and self-compassion skills were administered before the first session and after the last.

### Measures

#### Depressive Symptoms

The Dutch translation of the 20-item Beck Depression Inventory (BDI-II (Beck et al. 1996b); Dutch version: BDI-II-NL (Van der Does 2002) was used to assess depressive symptoms. This standardised questionnaire contains 21 items, scored on a 0–3 scale. The BDI-II has been validated in psychiatric outpatients. The internal consistency varies from 0.84 to 0.91 and the retest reliability ranged from 0.73 to 0.96 (Beck et al. 1996a; Wang and Gorenstein 2013).

#### Worry

The Dutch translation of the 16-item Penn State Worry Questionnaire (PSWQ (Meyer et al. 1990) was used to assess worry (responses are given on a 5-point scale). Possible range of scores is 16–80. The PSWQ has been demonstrated to have strong internal consistency (*α* of 0.95 at both test and retest) (Meyer et al. 1990).

#### Self-Compassion

The Dutch translation of the Self-Compassion Scale (SCS (Raes et al. 2011) was used to measure self-compassion skills of patients. The questionnaire consists of 26 items divided over three subscales: (1) self-kindness versus self-judgment, (2) common humanity versus isolation, and (3) mindfulness versus overidentification. On a scale of 1 to 5, participants indicate the extent to which they agree with statements such as “I try to be loving towards myself when I’m feeling emotional pain” (self-kindness), “When things are going badly for me, I see the difficulties as part of life that everyone goes through” (common humanity) and “When I'm feeling down I try to approach my feelings with curiosity and openness” (mindfulness). Internal consistencies of the different subscales vary from 0.75 to 0.81 and test-retest reliabilities vary from 0.80 to 0.93 (Raes et al. 2011). The SCS is sensitive to change in MBCT (Kuyken et al. 2010).

#### Mindfulness

Mindfulness skills were measured using the Five Facet Mindfulness Questionnaire (FFMQ-NL; (Baer et al. 2008), which has five subscales: observing, describing, acting with awareness, non-judging of inner experience and non-reactivity to inner experience. Internal consistencies of the different subscales vary from 0.72 to 0.93 (Baer et al. 2008). The FFMQ is sensitive to change in mindfulness-based interventions (i.e. MBSR, Carmody and Baer 2008).

### Data Analyses

The focus group interview was transcribed verbatim by RS and coded independently by RS and a senior researcher (HvR). These two coding researchers were trained in mindfulness, having successfully completed the teacher training program at the Radboudumc Centre for Mindfulness, as well as qualitative analysis, in a separate, intensive course. After the focus group, the codes were compared and discussed by the two coding researchers to identify possible discrepancies between codes until reaching consensus. This led to a coding scheme, to which new codes from the second focus group could be added. After the two focus groups, RS and HvR together with HJ, RM and AS grouped the codes into subthemes, and subthemes into themes for thematic analysis (Braun and Clarke 2006).

Emerging themes indicating suggestions for improvement from the first focus group were discussed with the original developers of MBCL (Van den Brink and Koster) and the MBCL program was adapted accordingly. Because the adaptations were major, we chose to offer the revised program for a second time to the original participants, of whom ten (71%) agreed to participate. The group was extended by three patients with recurrent depression who had not previously participated. A renewed focus group interview was conducted after the second course, also led by AS, accompanied by both teachers and RS, and lasting one-and-a-half hours. The interview was audio and videotaped, and transcribed verbatim (RS). RS and HvR coded the interview and discussed coding and thematic analysis with HJ, RM and AS. The themes emerging from the second interview were also discussed with the original developers Van den Brink and Koster and gave rise to a few additional, minor improvements of the program. The iterative process of analysis, adaptation and re-evaluation involving all parties in every step enabled the co-creation of an adapted format for MBCL.

To analyse the quantitative data, we conducted repeated measures ANOVA on all (sub)scales using SPSS 20. We report the findings of the two subsequent groups separately: the first analysis containing all novices to compassion training and the second one containing three novices in addition to ten second-time participants.

## Results

### First Focus Group Interview

Facilitators, i.e. elements of the program that were considered helpful by the participants, could be grouped into five themes, of which the three most salient ones are described in Table 2.

**Table 2:** Main facilitating programme components

Facilitators	Description
Didactic teaching	Evolutionary development and universality of the three emotion regulation systems Addressing and explanation of the back draft effect as a possible occurrence
Compassion practices	The explicit focus on self-compassion development in the practices, as well as on obstacles to self-compassion (inner bully/critic)
Embodiment teacher	Consistent mild attitude/responsiveness of the teachers Practical translation of compassion in daily life

#### Facilitators

##### Didactic Teaching

This theme was made up from theoretic elements in the content of the MBCL, such as theory on the evolution and universality of the human brain, theory on the three emotion regulation systems (threat, hunt and rest and digest system) and information regarding the back draft effect. Participants reported being helped by being reminded of the universality of the brain. Also, knowledge of the three systems gave them a reference point for practice.

The explanation, I found so illuminating. Which system you automatically step into. It starts with your brain, so enlightening. … It’s applicable to every brain and that is also comforting.

By addressing the back draft effect and its possible occurrence beforehand, participants were able to allow their emotions to be overwhelming for a while: they could accept it as a natural step in their process instead of taking it to mean something was wrong.

### Compassion Practices

Specific practices that were appreciated during the sessions and as homework included the safe haven and the compassionate body scan. As homework or more informal practice, writing a compassionate letter, keeping a diary and paying specific attention to the inner bully or critic were mentioned. This last practice involved practicing mindfulness of the primarily judgmental, critical voice or thought that often pops up for patients, helping them to discover when this voice is most present (i.e. supported by recognizing which system is active: threat, hunt or soothe) and what attack it will usually go for:

The inner bully was addressed extensively and that was a real eye-opener for me, …very recognizable as one of the major causes of distress. And by naming it and looking at it more compassionately, it got a place for me and it is a less dominant factor in my life than before.

### Teacher Embodiment of Compassion to Self and Others

Embodiment and compassion to self and others modelling by the teacher were considered very helpful by the participants:

It’s just that they [the teachers] also shared their own struggles with things that are difficult, so you’re really investigating this together.

Though many participants commented that the MBCL was confrontational and they suffered the back draft effect, most also indicated that going through this stage and cultivating an attitude with which the suppressed emotions could be approached instead of avoided, was what really propelled the development of compassion to self and others. The ability to approach seemed to be aided substantially by the teachers’ response to what participants shared in the group:

It really connects with me … when you listen to what people are sharing and what reactions you as instructors have to that. That is what’s helped me most of all … that translation ... in a safe group, which I found the most important, I’ve really experienced that.

### Peer Support

Group exchanges on (home) practices and experiences was also considered helpful by most participants:

You’re in a very democratic process of struggling together with what it is, and err yes that helps (…) especially when you’re depressed a lot, you think: o help, I’m so pathetic, and here you come into this group and yes, you’re in it together.

### Structure of the Training

In terms of the duration of the training, most participants indicated that eight sessions were good to get fully immersed in the program, though some thought that 10–12 sessions would be even better. Because of the amount of theory to address in the program, the number of practices to get acquainted with and importantly, the complexity of the subject, coming together once every other week instead of every week was highly appreciated by the participants.

#### Barriers

Barriers, i.e. elements of the program that were considered unhelpful by the participants, could be grouped into two themes (see Table 3).

**Table 3:** Main barriers in programme components

Barriers	Description
Compassion development	Being confronted with one’s lack of self-compassion as well as lack of compassion for others The resurfacing of old pain, i.e. the back draft effect
Structure of the training	Lack of structure Lack of core practice Too many practice options Unappealing course folder: language/volume

### Compassion Development

In general, participants commented on the MBCL being confrontational. Almost all participants reported struggling with the back draft effect during practices: being overwhelmed by old pain resurfacing now that they were allowing themselves to practice with approaching instead of avoiding hard thoughts and emotions. Also, they reported being confronted by how difficult it was to be kind to themselves, and how this realization grieved them.

… to look at myself more compassionately, has (…) made me confront a whole bunch of things that (…) are hard. I’ve found that (…) the price of this training, that it wasn’t easy for me, (…) it’s something to take into account.

Though they were helped by the theory on the back draft effect and the teachers’ approach of the subject, they stated they would have appreciated explanation of this phenomenon earlier in the program and also to have it addressed recurrently:

For me, it would have been easier if it had been addressed in some form in each session.

### Structure of the Training

Participants mentioned the program lacked a clear structure. For some participants, the main focus did not become obvious until session 4 or 5:

Perhaps followed by a summary and a reference of what the next step was going to be, what will we do next time and why is that a logical step considering what we’ve done before.

You [the teacher] returned to the soothing system and I thought: ah, finally, that’s what it’s about; I’ve been waiting five sessions to hear that.

In line with participants commenting on the lack of clear structure, they also found a lack of a core practice: each session brought a new practice and practices were seldom revisited in the curriculum. Also, the number of options to choose from for home practice seemed to be confusing to participants.

I do see the advantage of having a large shop in which to choose what I like, but for me I tend to walk past the shop when it’s like that.

It was not clear what should be practiced from session to session and participants were insecure about what was obligatory and what was non-obligatory:

Now everyone had done something else, so you can’t share so much how you’ve struggled with a particular practice.

It would have helped me to know which exercises are specific for this session, and then to be offered the rest as added options, in case you want to do more.

One of the participants mentioned that especially when you are depressed, your ability to make even relatively small decisions such as what to practice is compromised:

A characteristic of depressed people is their difficulty with making decisions.

Participants also found the content of the course folder much too dense, and the language unappealing and dry:

I’ve really had words which I had to look up on the Internet.

It was a difficult, heavy read for me.

Some attributed their difficulties with the practice to the fact that they could not place the program in a mindfulness framework; they had expected more common ground between MBCT and MBCL.

Several participants mentioned having difficulty with the content of the Metta or befriending practice in MBCL. Some found it hard to feel compassion for others in general, and some would have preferred the program to attend to self-compassion longer before moving on to compassion for others:

Yes, … it’s finally your turn and then you already had to transfer to someone else and then I thought, yeah but I don’t feel like doing that at all, I am finally on a roll with myself so that can wait a couple of sessions. I’m not saying to take it out of the program but it’s very liberating that you’re allowed to just have compassion with yourself for a change.

### Adaptations to the MBCL Program After the First Focus Group Interview

The possible improvements that emerged from analysis of the first interview and subsequent discussion with the developers were used to adapt the MBCL. Considering the impact of the back draft effect on the participants, we decided to address the possible occurrence of this effect earlier in the program (session 2 instead of 4) and check up on this more regularly and explicitly throughout the entire program. Allocating more time to the back draft effect meant we had to sacrifice some time to discuss the inner critic though.

Next, we addressed the curriculum practicalities of MBCL: we restructured the curriculum over the sessions according to the MBCT format more explicitly. Especially sessions 3 and 4 were restructured to resemble MBCT’s format of addressing craving and aversion, respectively. The course folder was highly simplified and the style of writing was made more personal. Given the comments on the overwhelming amount of homework options, we simplified the program by cutting down the number of exercises. An exercise on imagining a compassionate companion was removed from the program as a separate practice, though it was introduced in modified manner at the beginning of the Metta practice. The simplification of practices enabled us to introduce the Soften-Soothe-Allow exercise from Germer (2009), which guides practitioners very gently and gradually to being with an unpleasant experience, as well as providing support for the back draft effect. We slowed down the transition from self-compassion to compassion for others, essentially dividing the program into four sessions focusing on self-compassion and four sessions focusing on both self-compassion and compassion for others.

### Second Focus Group Interview

Thematic analysis of codes found in the second focus group interview largely supported the adaptations made to the program. Participants reported being very happy with the extra space allowed for the back draft effect. Also, Soften-Soothe-Allow (Germer 2009) was indeed mentioned as particularly helpful support in dealing with this effect. Participants also indicated being much more satisfied with the number of homework options offered the second time around, though they would still like to reduce the total number. The exercises that were removed from the program did not seem to be missed. Due to the simplification of the program and reduction in practice options, participants felt that there was more room for inquiry and exchange than during the first course. Even more than in the first evaluation, they commented that the honest and often vulnerable sharing of the teachers of their own experiences was a good model of a compassionate attitude to both self and others:

The second time we took more time to exchange experiences in the group on what was encountered, if you were managing to practice or not, how you dealt with yourself. For me, I feel that taught me most of all, because you [the teachers] were very consistent in being mild.

Also, the additional time for enquiry encouraged the exchange between participants. When participants could not work with compassion to self or others in a certain difficult experience, it was helpful to hear from others how they did just that:

Yes, okay, it’s explained, but still.. and then someone would say something that would make it click with me, so what they [the teachers] said became clear through her [participant] story.

In terms of practicalities, it seemed the course folder had been condensed too much: participants commented on elements they now missed, such as the background information on the inner critic or bully:

In the first course, the inner bully was addressed extensively and that was a huge eye-opener for me like that is so recognizable as one of the major causes of all the unrest, and by naming it and looking at it from a compassionate viewpoint, it has a less dominant role in my life than before.

We adapted the course folder accordingly. For an overview of the entire adapted MBCL program, see Appendix I in the Supplemental Materials.

### Preliminary Effectiveness

The study was set up as a co-creation and feasibility study, not as a randomised controlled trial. Therefore, this paragraph only contains preliminary indications of effectiveness, which must be interpreted as such. For the first course, we analysed *N* = 13 as one of the participants failed to hand in the post-measurement. For the remaining 13 participants, no changes in outcome were found with regard to depressive symptoms or worry. With the exception of the subscale *Observe*, mindfulness skills did not change (see Table 4 and Table 5). Self-compassion overall did change (Cohen’s *d* = 0.56), particularly the subscale *Common Humanity* (Cohen’s *d* = 0.54).

**Table 4:** Quantitative results MBCL from pre to post per group using a repeated measures ANOVA

Group 1 (*N* = 14)							Group 2 (*N* = 13)^b^						
Scale	Subscale	Mean		CI (diff)	*p*	*d*	Scale	Subscale	Mean		CI (diff)	*p*	*d*
		Pre (SD)	Post (SD)						Pre (SD)	Post (SD)			
BDI-II	Depression	13.14 (9.9	10.79 (7.7)	−1.90–6.61	.253	0.26	BDI-II	Depr	21.13 (12.4)	13.00 (12.1)	4.60–11.66	.001^b^	0.66
PSWQ^a^	Worry	43.69 (6.7)	42.15 (6.7)	−1.97–5.05	.359	0.23	PSWQ^c^	Worry	43.14 (8.3)	45.57 (8.2)	−9.16–4.30	.411	0.29
FFMQ	Mindfulness	80.64 (8.8)	83.79 (9.1)	−8.13–1.84	.196	0.35	FFMQ	Mindf	79.38 (17.2)	81.63 (14.5)	−8.12–3.62	.395	0.14
	Observe	15.93 (1.1)	17.00 (1.5)	−1.87–−0.27	.013^a^	0.81		Obs	15.50 (3.0)	15.86 (2.6)	−2.32–1.57	.662	0.13
	Describe	20.00 (4.2)	20.21 (3.8)	−1.85–1.42	.782	0.05		Descr	18.88 (4.9)	17.88 (4.4)	−0.84–2.84	.240	0.21
	Act with awareness	15.36 (2.2)	15.93 (3.1)	−2.31–1.16	.489	0.21		Act	16.25 (4.4)	16.63 (3.3)	−1.92–1.17	.584	0.10
	Non-judgment	14.14 (2.7)	15.14 (4.0)	−2.52–0.52	.179	0.29		Non-judg	14.25 (3.0)	16.63 (3.9)	−4.32–−0.43	.023^b^	0.68
	Non-reactivity	15.21 (2.7)	15.50 (2.8)	−1.86–1.29	.702	0.11		Non-reac	15.50 (4.2)	14.63 (3.2)	−1.42–1.17	.826	0.23
SCS^a^	Self-compassion	23.09 (5.2)	26.25 (6.0)	−6.21–−0.11	.043^a^	0.56	SCS^b^	Self-comp	22.31 (7.8)	25.26 (8.0)	−5.81–−0.09	.045^b^	0.37
	Self-kindness	4.03 (1.2)	4.60 (1.4)	−1.14–0.03	.059	0.44		Self-kind	3.81 (1.6)	4.63 (1.5)	−1.53–−0.09	.032^b^	0.53
	Self-judgment	4.33 (1.1)	4.04 (1.3)	−0.90–1.48	.606	0.24		Self-judg	4.00 (1.3)	4.41 (1.4)	−2.51–1.70	.662	0.30
	Common humanity	4.10 (1.3)	4.75 (1.1)	−1.28–−0.03	.041^a^	0.54		Comm hum	3.72 (1.6)	4.14 (1.7)	−1.26–0.43	.281	0.25
	Isolation	4.56 (1.1)	4.02 (1.3)	−0.80–1.87	.397	0.49		Isol	4.75 (1.7)	3.81 (1.6)	−1.76–3.63	.438	0.57
	Mindfulness	4.51 (1.0)	4.90 (0.9)	−0.80–0.02	.058	0.41		Mindf	4.25 (1.4)	4.60 (1.4)	−0.70–0.01	.054	0.25
	Overidentification	4.67 (1.1)	3.94 (1.1)	−0.49–1.95	.218	0.69		Overid	4.72 (1.1)	3.70 (1.1)	−0.68–2.74	.197	0.93

^a^ *N* = 13, one post-measurement missing

^b^ *N* = 9, one pre and three post-measurements missing (all female)

^c^ *N* = 8

**Table 5:** Pre-post scores group 2 on overall scales

	Doubles (*N* = 10)^a^ Mean		Novices (*N* = 3) Mean	
	Pre (sd)	Post (sd)	Pre (sd)	Post (sd)
BDI-II	20.6 (11.9)	10.4 (11.6)	25 (9)	22 (1.4)
PSWQ	42.8 (7.8)	41.7 (7.1)	54 (2)	59 (0)
FFMQ	79.8 (15.2)	85.7 (12.8)	70 (14.4)	72.5 (17.7)
SCS	24.2 (6.5)	28.5 (7.5)	15.3 (4.5)	18.2 (1.5)

^a^ *N* = 6, one pre and three post-measurements missing (all female)

For the second course, we analysed *N* = 9 participants as one did not hand in the pre-measurement, and three others did not hand in the post-measurement. In group 2, a significant reduction of depressive symptoms was found (Cohen’s *d* = 0.66), but no changes in worry. Except for *Non-judgment* (Cohen’s *d* = 0.68), no changes in mindfulness skills were found, but overall self-compassion (Cohen’s *d* = 0.37) again improved, particularly *Self-kindness* (Cohen’s *d* = 0.53), *Isolation* (Cohen’s *d* = 0.57) and *Overidentification* (Cohen’s *d* = 0.93).

## Discussion

The results of our pilot study on the co-creation and feasibility of a compassion training for patients with recurrent depression are encouraging. The attendance rate was very high for both courses. In contrast to the first version of the course, participants reported being very satisfied with the adapted program. In accordance with this, the reduction of depressive symptoms was greater in the second course than in the first. Pre-scores on the BDI-II of the second group were on average higher than in the first group; this difference was, however, the strongest with the novices in the second course. In both groups, improvements in self-compassion were found, both overall and in several subscales, with effect sizes ranging from small to large. This may indicate that MBCL delivered what it is designed to do: improve (self)compassion skills. No reduction was found in worry, nor improvement in mindfulness skills overall. This might be explained by the fact that all participants already participated in an MBCT course before taking part in the compassion training. It is known that MBCT results in a reduction of worry and an improvement of mindfulness skills (Van Aalderen et al. (2012). However, as the study sample was small, it is obvious that these quantitative results should be interpreted with the utmost caution: especially the reduction in depressive symptoms in the second group may also have been due to the ‘double dosage’ of MBCL that most participants in that group had received by then. In general though, these results are congruent with the hypothesis proposed by Koster and Van den Brink (2012) that self-compassion skills can be significantly increased by explicitly training them. It seems that even for patients who previously attended MBCT explicit compassion training is of added value.

By inviting frequent attendees of reunion meetings to participate in this pilot study, our selection procedure was probably biased in favour of patients had followed MBCT quite some time ago: they may have experienced a decline in experienced effects from MBCT and thus have been motivated for a follow-up program. This may have overestimated our results. The acceptability and effectiveness might be less pronounced in less motivated participants, or if we had recruited participants immediately after MBCT. In addition, we have very little data on the proportion and characteristics of patients who declined our offer to participate; these patients may be especially interesting in terms of barriers to the MBCL program.

However, we were also very happy to be able to work with highly motivated participants (‘lead users’) in order to get informed feedback on the MBCL to improve the curriculum. As we received ample feedback on how to improve the first version of the course and were able to evaluate the adapted version of the programme with almost all original attendees, we are confident we now have a program suitable for this population.

To further examine the effectiveness of MBCL, a properly powered randomised controlled trial should be conducted. This may help to answer questions about (a) the possible added value of an explicit training in compassion for patients with recurrent depression who previously participated in MBCT and (b) whether compassion training should be a follow-up to MBCT, or whether it might be valuable as an adapted/stand-alone intervention in this population.

In conclusion, MBCL as an intervention for patients suffering from recurrent depressive symptoms appears to be feasible and acceptable, and the preliminary results on the effectiveness of the program in terms of reducing depressive symptoms and increasing self-compassion are promising. The results of this pilot study indicate that the cultivation of self-compassion might deserve more attention in this population than it currently gets. Though this is a small and uncontrolled feasibility study and our findings are preliminary, the next step should be a properly powered, randomised controlled trial.

## Electronic Supplementary Material

ESM 1 (DOCX 12 kb)

## References

[CR1] Baer RA, Smith GT, Lykins E, Button D, Krietemeyer J, Sauer S, Walsh E, Duggan D, Williams JM (2008). Construct validity of the five facet mindfulness questionnaire in meditating and nonmeditating samples. Assessment.

[CR2] Bartels-Velthuis, A. A., Schroevers, M. J., van der Ploeg, K., Koster, F., Fleer, J., & van den Brink, E. (2016). A mindfulness-based compassionate living training in a heterogeneous sample of psychiatric outpatients: a feasibility study. *Mindfulness, 7*(809). doi:10.1007/s12671-016-0518-8. 10.1007/s12671-016-0518-8 PMC4923083 27429664

[CR3] Beck AT, Steer RA, Ball R, Ranieri WF (1996). Comparison of Beck Depression Inventories-IA and-II in psychiatric outpatients. Journal of Personality Assessment.

[CR4] Beck AT, Steer RA, Brown GK (1996). Beck depression inventory-II.

[CR5] Braun V, Clarke V (2006). Using thematic analysis in psychology. Qualitative Research in Psychology.

[CR6] Carmody J, Baer RA (2008). Relationships between mindfulness practice and levels of mindfulness, medical and psychological symptoms and well-being in a mindfulness-based stress reduction program. Journal of Behavioral Medicine.

[CR7] First M, Gibbon M, Spitzer R, Williams J (1996). User’s guide for the structured interview for DSM-IV axis I disorders—research version (SCID-I, version 2.0, February 1996 final version).

[CR8] Franke N, Von Hippel E, Schreier M (2006). Finding commercially attractive user innovations: a test of lead-user theory. Journal of Product Innovation Management.

[CR9] Germer CK (2009). The mindful path to self-compassion: freeing yourself from destructive thoughts and emotions.

[CR10] Gilbert P (2009). Introducing compassion-focused therapy. Advances in Psychiatric Treatment.

[CR11] Gilbert P, Procter S (2006). Compassionate mind training for people with high shame and self-criticism: overview and pilot study of a group therapy approach. Clinical Psychology & Psychotherapy.

[CR12] Gilbert P, McEwan K, Mitra R, Franks L, Richter A, Rockliff H (2008). Feeling safe and content: a specific affect regulation system? Relationship to depression, anxiety, stress, and self-criticism. The Journal of Positive Psychology.

[CR13] Gilbert P, McEwan K, Gibbons L, Chotai S, Duarte J, Matos M (2012). Fears of compassion and happiness in relation to alexithymia, mindfulness, and self-criticism. Psychological Psychotherapy.

[CR14] Hardeveld F, Spijker J, De Graaf R, Nolen W, Beekman A (2010). Prevalence and predictors of recurrence of major depressive disorder in the adult population. Acta Psychiatrica Scandinavica.

[CR15] Kuyken W, Watkins E, Holden E, White K, Taylor RS, Byford S, Evans A, Teasdale JD, Dalgleish T (2010). How does mindfulness-based cognitive therapy work?. Behaviour Research and Therapy.

[CR16] Kuyken W, Warren FC, Taylor RS, Whalley B, Crane C, Bondolfi G, Hayes R, Huijbers M, Ma H, Schweizer S, Segal Z, Speckens A, Teasdale JD, Van Heeringen K, Williams M, Byford S, Byng R, Dalgliesh T (2016). Efficacy of mindfulness-based cognitive therapy in prevention of depressive relapse: an individual patient data meta-analysis from randomized trials. Journal of the American Medical Association: Psychiatry.

[CR17] Lusch RF, Vargo SL, O’Brien M (2007). Competing through service: insights from service-dominant logic. Journal of Retailing.

[CR18] MacBeth A, Gumley A (2012). Exploring compassion: a meta-analysis of the association between self-compassion and psychopathology. Clinical Psycholical Review.

[CR19] Meyer TJ, Miller ML, Metzger RL, Borkovec TD (1990). Development and validation of the penn state worry questionnaire. Behaviour Research and Therapy.

[CR20] Mueller TI, Leon AC, Keller MB, Solomon DA, Endicott J, Coryell W, Warshaw M, Maser JD (1999). Recurrence after recovery from major depressive disorder during 15 years of observational follow-up. American Journal of Psychiatry.

[CR21] Neff K (2003). The development and validation of a scale to measure self-compassion. Self and Identity.

[CR22] Neff K, Germer C (2013). A pilot study and randomized controlled trial of the mindful self-compassion program. Journal of Clinical Psychology.

[CR23] Piet J, Hougaard E (2011). The effect of mindfulness-based cognitive therapy for prevention of relapse in recurrent major depressive disorder: a systematic review and meta-analysis. Clinical Psychological Review.

[CR24] Raes F, Pommier E, Neff KD, Van Gucht D (2011). Construction and factorial validation of a short form of the self-compassion scale. Clinical Psychological Psychotherapy.

[CR25] Segal ZV, Williams JMG, Teasdale JD (2012). Mindfulness-based cognitive therapy for depression.

[CR26] Sheehan DV, Lecrubier Y, Sheehan KH, Amorim P, Janavs J, Weiller E, Herqueta T, Baker R, Dunbar GC (1998). The Mini-International Neuropsychiatric Interview (MINI): the development and validation of a structured diagnostic psychiatric interview for DSM-IV and ICD-10. Journal of Clinical Psychiatry.

[CR27] Solomon DA, Keller MB, Leon AC, Mueller TI, Lavori PW, Shea MT, Coryell W, Warshaw M, Turvey C, Maser JD, Endicott J (2000). Multiple recurrences of major depressive disorder. American Journal of Psychiatry.

[CR28] Strauss C, Cavanagh K, Oliver A, Pettman D (2014). Mindfulness-based interventions for people diagnosed with a current episode of an anxiety or depressive disorder: a meta-analysis of randomised controlled trials. PloS One.

[CR29] Van Aalderen J, Donders A, Giommi F, Spinhoven P, Barendregt H, Speckens A (2012). The efficacy of mindfulness-based cognitive therapy in recurrent depressed patients with and without a current depressive episode: a randomized controlled trial. Psychological Medicine.

[CR30] Van Aalderen JR, Donders ART, Peffer K, Speckens AE (2015). Long-term outcome of mindfulness-based cognitive therapy in recurrently depressed patients with and without a depressive episode at baseline. Depression and Anxiety.

[CR31] Van den Brink E, Koster F (2012). Compassievol leven: van mindfulness naar heartfulness.

[CR32] Van den Brink E, Koster F (2015). Mindfulness-based compassionate living: a new training programme to deepen mindfulness with heartfulness.

[CR33] Van der Does A (2002). BDI-II-NL. Handleiding. De Nederlandse versie van de beck depression inventory.

[CR34] Vargo SL, Lusch RF (2004). Evolving to a new dominant logic for marketing. Journal of Marketing.

[CR35] Wang Y-P, Gorenstein C (2013). Assessment of depression in medical patients: a systematic review of the utility of the Beck Depression Inventory-II. Clinics.

