# Supplementary material for: The Co-creation and Feasibility of a Compassion Training as a Follow-up to Mindfulness-Based Cognitive Therapy in Patients with Recurrent Depression
Source: Mindfulness (N Y). 2017 Aug 5;9(2):412–22. doi: 10.1007/s12671-017-0783-1 (PMC5866843; doi:10.1007/s12671-017-0783-1)
Supplement: Supplementary file 1 — (DOCX 12 kb) [file 12671_2017_783_MOESM1_ESM.docx]

**Supplemental Materials**

# APPENDIX I

# Summarized description adapted MBCL

Session 1

Introduction to compassion

Practice : Compassionate body scan + inquiry

Psycho-education on the three systems, connected to stress reactions of fight/flight/freeze

Practice: Safe haven

Compassionate breathing

Session 2

Arriving

Practice: Soften-soothe-allow + inquiry

Inquiry home practice

Theoretical information on back draft effect and obstacles to self-compassion in general

Practice: Metta (only benefactor) + inquiry

Homework suggestions

Compassionate breathing space + poem

Session 3

Arriving

Practice: Compassionate movement (lying down) + inquiry

Inquiry home practice

Theoretical information on the inner bully (part 1)

Exercise with scheme therapy questionnaires to identify compensation strategies

Practice: Metta (benefactor and self) + inquiry

Homework suggestions

Compassionate breathing space

Session 4

Arriving

Practice: Soften-soothe-allow + inquiry

Inquiry home practice

Theoretical information on recognizing patterns; the inner bully (part 2)

Practice: Metta (benefactor and self) + inquiry

Homework suggestions

Compassionate breathing space with emotional pain

Session 5

Arriving

Practice: Compassionate movement exercises (standing) + inquiry

Inquiry home practice

Practice: Metta (benefactor, self and important other)

Homework suggestions

Compassionate breathing space with emotional pain

Session 6

Arriving

Practice: Metta (benefactor, self, important other and neutral person) + inquiry

Inquiry home practice

Interactive psycho-education on common humanity:

Writing a compassionate letter to yourself

Practice: Compassionate walking

Homework suggestions

Compassionate breathing space and poem

Session 7

Arrival

Practice: Soften-soothe-allow + inquiry

Inquiry home practice

Practice: Forgiveness towards others

Practice: Metta (benefactor, self, important other, neutral person and difficult other or some aspect(s) of yourself you struggle with) + inquiry

Homework suggestions

Compassionate breathing space

Session 8

Arrival

Practice: Metta (benefactor, self, important other, neutral person, difficult other or some aspect(s) of yourself you struggle with and all beings) + inquiry

Inquiry home practice

Group exchange on the future: what is your plan?

Ending the course through symbols participants were invited to bring, what do you wish for yourself and others?

Compassionate breathing space and poem
